# Malnutrition and Childhood Disability in Turkana, Kenya: Results from a Case-Control Study

**DOI:** 10.1371/journal.pone.0144926

**Published:** 2015-12-21

**Authors:** Hannah Kuper, Velma Nyapera, Jennifer Evans, David Munyendo, Maria Zuurmond, Severine Frison, Victoria Mwenda, David Otieno, James Kisia

**Affiliations:** 1 International Centre for Evidence in Disability, London School of Hygiene & Tropical Medicine, London, United Kingdom; 2 Kenya Red Cross Society, Nairobi, Kenya; 3 Clinical Research Department, London School of Hygiene & Tropical Medicine, London, United Kingdom; 4 CBM Kenya, Nairobi, Kenya; 5 Department of Disease Control, London School of Hygiene & Tropical Medicine, London, United Kingdom; TNO, NETHERLANDS

## Abstract

**Background:**

Children with disabilities may be particularly vulnerable to malnutrition, as a result of exclusions and feeding difficulties. However, there is limited evidence currently available on this subject.

**Methods:**

A population-based case-control study was conducted in Turkana County, Kenya, between July and August 2013. Key informants in the community identified children aged 6 months to 10 years who they believed may have a disability. These children were screened by a questionnaire (UNICEF-Washington Group) and assessed by a paediatrician to confirm whether they had a disability and the type. Two controls without disabilities were selected per case: A sibling control (sibling nearest in age) and a neighbourhood control (nearest neighbour within one year of age). The caregiver completed a questionnaire on behalf of the child (e.g. information on feeding, poverty, illness, education), and anthropometric measures were taken. We undertook multivariable logistic and linear regression analyses to estimate the relationship between disability and malnutrition.

**Results:**

The study included 311 cases with disabilities, 196 sibling controls and 300 neighbour controls. Children with disabilities were more likely to report a range of feeding difficulties. They were 1.6–2.9 times more likely to have malnutrition in comparison to neighbour controls or family controls, including general malnutrition (low weight for age), stunting (low height for age), low body mass index (BMI) or low mid upper arm circumference (MUAC) for age. Children with disabilities were almost twice as likely to have wasting (low weight for height) in comparison to neighbour controls (OR = 1.9, 95% CI 1.1–3.2), but this difference was not apparent compared with siblings (OR = 1.5, 95% CI 0.8–2.7). Children with disabilities also faced other exclusions. For instance those aged 5+ were much more likely not to attend school than neighbour controls (OR = 8.5, 95% CI 4.3–16.9).

**Conclusions:**

Children with disabilities were particularly vulnerable to malnutrition, even within this area of food insecurity and widespread malnutrition. Efforts need to be made to include children with disabilities within food supplementation programmes, and school based programmes alone may be inadequate to meet this need. Exclusion of children with disabilities from education is also a priority area to be addressed.

## Background

The World Report on Disability estimates that there are approximately 93 million children aged 0–14 years living with “moderate or severe disability” equating to one in twenty children globally (5·1%), while 0.7% have a severe disability.[1] Childhood disability is particularly common in low and middle income countries (LMICs), where malnutrition is a leading cause of childhood death.[2]

Malnutrition may be linked to childhood disability. This relationship is multifaceted, as malnutrition may be both a cause and effect of disability, and the relationship is likely to operate in different ways at different stages in life.[3] Children with disabilities may experience difficulties in feeding, poorer absorption of nutrition or neglect, which can increase their vulnerability to malnutrition.[4,5] Malnutrition at a young age may also lead to the development of disability, through insufficiency in micro/macronutrients, a high concentration of anti-nutrients or by increasing vulnerability to developmental delay.[5] Older children with disabilities may be less likely to attend school and therefore are not able to benefit from school feeding programmes. Few studies have addressed the relationship between disability and malnutrition and the existing evidence is inconclusive. Some studies have shown a positive association between disability and malnutrition,[6] while others have reported mixed findings,[7] or have demonstrated a link only among children with learning difficulties or cerebral palsy.[8,9]

If a relationship exists between disability and malnutrition then this vulnerability may become heightened during periods of humanitarian crisis.[10] One such region is Turkana County in north western Kenya. Turkana is the poorest area in Kenya, it is extremely arid and has experienced poor food security for many years.[11,12] It is not clear, however, what the nutritional situation is for children with disabilities and whether they are included in food distribution programmes in the area. One such programme is the school feeding programme, which provides maize porridge daily to children attending school.

The primary aim of the study was therefore to assess whether there was an association between disability and malnutrition and whether children with disabilities were included within food distribution programmes, in Turkana County Kenya. We also aimed to investigate the associations of childhood disability with education, poverty and health in order to allow consideration of potential pathways for any relationship between disability and malnutrition.

## Materials and Methods

The focus of the research was the Turkana Central and Loima sub- counties in Turkana County, Kenya. The fieldwork was undertaken between July and August, 2013. The study design was a population-based case-control study.

### Selection of children with disabilities

Children with disabilities aged 6 months to 10 years were identified in the community through key informants familiar with the target area (community health workers, Kenyan Red Cross volunteers, representatives from disabled people’s organisations). The key informants were selected geographically so that they covered all the communities within the two sub-counties. The informants underwent one day of training including information on disability sensitisation, methods for case detection and identification of different impairments. They then returned to their village where they identified children who potentially had a disability over a two week period. All children with disabilities identified by key informants in the target area were included in the study. This method has been previously validated in Bangladesh for identifying children with disabilities and estimating the prevalence and types of disability.[13,14]

A paediatrician and an interviewer visited each potential case identified by the key informants to ascertain whether or not the child had a moderate to severe impairment. The child was assessed using the Washington Group-UNICEF childhood disability questionnaire, which included 15 questions and asked each child’s parent/guardian to report on difficulties experienced by the child in any of 12 domains (seeing, hearing, walking, self-care, communication/ comprehension, learning, emotions, behaviour, attention, coping with change, relationships, playing). For each question the response options were “no difficulty”, “some difficulty”, “a lot of difficulty” or “cannot do at all”. A child was considered to have a moderate to severe impairment if the response was “a lot of difficulty” or “cannot do” for at least one question.

The paediatrician then examined the child to ascertain the type, severity and cause of impairment as well as the child’s rehabilitation and medical needs. The paediatricians classified the types of impairment as follows:

- Physical impairment (determined through physical examination)
- Epilepsy (questionnaire)
- Visual impairment (measuring vision with E chart for children 5–10, or through fixation on objects for children <5)
- Hearing impairment (questions, response to noise and examination with otoscope)
- Intellectual impairment (professional opinion of paediatrician)

Children could be recorded as having multiple impairments.

### Selection of control subjects

Two control subjects aged 6 months to 10 years were selected for each child with disability (case). The first was the neighbour control to allow assessment as to whether children with disabilities were more likely to be malnourished compared to other children in the community. The neighbour control selected was a child nearest in age (within 12 months) living closest to the case and preferentially of the same sex. A child was not eligible to be a neighbour control if he/she lived in the same household as a case. The second control was a household control to indicate whether children with disabilities were more likely to be malnourished compared to other children in their household. The household control selected was the sibling closest in age living in the same household as the case.

Potential controls also undertook the Washington Group-UNICEF questionnaire and were examined by the paediatrician to ensure that they did not have a moderate to severe impairment (if so, they were included as a case).

### Questionnaires

The caregivers of the child (whether case or control) were interviewed in their homes with a semi-structured questionnaire, using standardised questions where available. This included questions on:

- socio-demographics (age, gender, household characteristics, poverty measures) (Based on tools of Living Standards Measurement Survey) [15]
- schooling (attendance, grade, absentees)
- water and sanitation (source of water, type of toilet, handwashing)[15]
- health of the child (vaccination status, vitamin A and de-worming, illness in last 2 weeks, health seeking behavior) [15]
- feeding habits and difficulties (who feeds child, difficulties on feeding with coughing, vomiting, chewing or refusing food)[6]
- inclusion in feeding programmes

Several items were included to measure poverty including: asset ownership (e.g. mobile phone, radio, bicycle, motorcycle, table, candles, lamps, solar panel and cupboard), receipt of aid in last 3 months, whether largest household expenditure was on food, and education of head of household. A food frequency questionnaire was also administered that was developed locally to reflect the typical foods eaten by participants on a regular basis (cereal products, white tubers roots and plantains, vitamin A rich foods, other vegetables, other fruits, meat, eggs, fish, dairy, oils/fats, baked goods, tea). It asked about food groups consumed in the last 24 hours and over the last 7 days at both the household level and for the individual child.

### Anthropometry

Anthropometric measures were taken by trained field workers. The measurements taken included:

- Weight—measured with minimal light clothing on, using the electronic UNISCALE (mother and child scale) and recorded to the nearest 0.1kg.
- Height/length—measured for children bareheaded and barefooted using wooden height boards and recorded to the nearest 0.1cm. For children under the age of two years recumbent length was taken and for those over two years height was taken while standing upright. Height rods with a marking tape were used to assist in determining measurement for taller children
- Mid Upper Arm Circumference (MUAC)—measured using child tapes and recorded to the nearest 0.1cm
- Arm-span—measured from the tip of the middle finger of one arm to the tip of the middle finger of the other arm with the arms outstretched at right angles to the body and recorded to the nearest 0.1 cm.
- Arm-length–measured from the tip of the humerus bone to the tip of the middle finger of the left arm and recorded to the nearest 0.1 cm.
- Tibia length—measured from the knee joint to the ankle joint and recorded to the nearest 0.1 cm.

Each measurement was repeated three times.

### Training of field staff and pilot study

The questionnaires were assessed for local relevance and appropriateness and were also pilot tested where they were found to work well. All survey instruments were produced in English and then translated into Ng’aturkana and adapted to the local context. The translated questionnaires were reviewed by the enumerators during their training for authenticity and accuracy of translation.

Training for the field team in Turkana lasted approximately one week and covered both details of the questionnaire and interview techniques. The paediatricians underwent two days of training by an ophthalmologist and audiologist on the topics of visual and hearing impairment. The anthropometry team and paediatricians were given theoretical training and practical sessions over a period of 5 days.

### Data analysis

Data were double-entered in Kenya including range and consistency checks. All analyses were done in Stata version 12 (StataCorp, Texas).

Height, weight, mid-upper arm circumference, arm length and span and tibia length were calculated from the mean of three measurements. An asset score was derived through adding the number of assets owned.

The data were analysed to assess the prevalence of malnutrition and its correlates comparing cases to the two control groups, in turn.

First, we undertook simple descriptive tabulations comparing cases with neighbourhood and household controls. We undertook multivariable logistic and linear regression analyses to estimate the relationship between disability and anthropometry, socio-demographic characteristics, including age, sex, and poverty markers. Household characteristics were compared between cases and neighbourhood controls only (these did not differ between cases and sibling controls). These analyses generated odds ratios (logistic regression) and beta coefficients (linear regression) with 95% confidence intervals. Conditional logistic regression was not undertaken as the cases were not always matched to neighbour or sibling controls, and so instead we adjusted for the matching variables (age and sex).

Malnutrition was considered in terms of: general malnutrition (low weight for their age), stunting (low height for age), wasting (low weight for height), low BMI and low MUAC for age. The nutritional status of the children was compared to the WHO Child Growth Standards, by calculating standard deviation scores ("z-scores") using Stata macros for weight for age, height for age and body mass index (BMI) for age for all children and weight for height and MUAC for age for children 5 years and younger. [16] We excluded children with z-score values outside the recommended range (z-scores greater than 5 or 6/less than -5 or -6, depending on the measure). We defined "stunting” (low height for age), "general malnutrition” (low weight for age), "low BMI for age", “wasting” (low weight for height) and low MUAC for age as a z-score of -2 or less.

We reran the analyses using arm span instead of height, given that we anticipated that not all of the children with disabilities would be able to stand for the height measurement. Analyses were restricted to cases and controls where complete data for the relevant variables in the regression were available.

### Ethics

The project received ethical approval from Institutional Research and Ethics Committees from Moi Teaching and Referral Hospital in Kenya, and from the London School of Hygiene & Tropical Medicine, UK. Written informed consent was obtained from the primary caregiver of each child. The children included were under the age of 10, and so child assent was not formally obtained. A family member was present during the examination of each child. Children with disabilities or malnutrition requiring services were referred as appropriate.

## Results

The key informants identified 308 children with moderate to severe disabilities. A further 42 children were identified as potentially having a disability, but were deemed not eligible and excluded (not in the correct age group or impairment considered as mild). On examination, three controls were identified as cases and were included in the case group. In total, we included 311 cases, 196 sibling controls and 300 neighbour controls. We estimated that there were approximately 41,674 children aged <10 living in Turkana Central and Loima sub counties.[17] The minimum prevalence of moderate to severe disability in children in Turkana County was therefore 0.75% (95% CI 0.66–0.83%).

The average age of the cases was 2.8 years, with 43% below the age of 5, and two thirds were boys (Table 1). The cases and controls were relatively well matched with respect to age and sex. The 311 children with disabilities had 321 diagnoses as determined by the paediatrician (some had multiple impairments). The most common impairment group was physical impairment (42%), among whom 31% had cerebral palsy, 14% had rickets and 10% had muscular dystrophy. Intellectual impairment was responsible for 22% of diagnoses (n = 88), and included 20 children with Down’s syndrome. Epilepsy, hearing impairment and visual impairment were less common.

**Table 1:** Characteristics of children with disabilities and controls.

		Child with disabilities N (%) N = 311	Sibling control N (%) N = 196	Neighbour control N (%) N = 300
Sex	Male	201 (65%)	107 (57%)	177 (60%)
	Female	109 (35%)	80 (43%)	118 (40%)
Age	6 months-<2	26 (9%)	22 (12%)	26 (9%)
	2–4	96 (31%)	72 (38%)	101 (34%)
	5–7	114 (37%)	64 (34%)	125 (42%)
	8–10	70 (23%)	33 (17%)	48 (16%)
Mean Age		2.8 (0.9)	2.6 (0.9)	2.7 (0.9)
Type of impairment	Physical	172 (42%)		
	Epilepsy	56 (14%)		
	Visual	34 (8%)		
	Hearing	59 (14%)		
	Intellectual	88 (22%)		

Only 46 of the 311 children with disabilities had ever received rehabilitation (15%), including therapy/exercises (n = 14), assistive devices (n = 5), surgery (n = 5), advice (n = 5) or other services (n = 16). The majority of services received were at the hospital (n = 30). For those children who had not sought treatment the most common reasons were: lack of awareness (34%), lack of money (33%), lack of perceived need (18%) or lack of transport (4%). The apparent cause of disability was reported for 267 children, and included congenital (67%), illness (13%), birth (6%) and trauma (5%).

Children with disabilities were significantly more likely to have a female head of household, compared to neighbour controls (Table 2). Other household characteristics did not differ between cases and neighbour controls. Children with disabilities (aged 5+) were more likely not to be attending school than neighbour controls (OR = 8.5, 95% CI 4.3–16.9). Among those who did attend school, cases were more likely to be at a lower level in the education system, but were not more likely to miss days of school. Cases were also more likely not to attend school in comparison to their sibling without disabilities (OR = 3.5, 95% CI 1.6–8.0) and were at a lower level of education. All children attending school took part in the school feeding programme, which was not available to those not at school. There were no differences between cases and neighbour controls in health measures.

**Table 2:** Relationship of disability with socio-demographics, school attendance (children aged 5+) and health.

		Child with disabilities N (%)	Neighbour control N (%)	Age and sex adjusted OR (95% CI)
**Socio-demographic**				
Head of household	Adult male	250 (84%)	262 (90%)	Reference
	Adult female	46 (16%)	26 (9%)	1.9 (1.1–3.2)
	Child	0	2 (0.7%)	-
Education of household head	None/Non-formal	230 (81%)	230 (80%)	Reference
	Primary	41 (14%)	32 (11%)	1.3 (0.8–2.1)
	Secondary/above	14 (5%)	23 (8%)	0.6 (0.3–1.3)
Polygamous family	Yes	134 (45%)	131 (45%)	1.0 (0.7–1.3)
	No	163 (55%)	160 (55%)	Reference
Living in location for > = 1 year	Yes	284 (95%)	279 (96%)	1.0 (0.5–2.2)
	No	14 (5%)	13 (4%)	Reference
Main source of income	Sale of firewood	83 (28%)	78 (27%)	Reference
	Petty trade	45 (15%)	50 (17%)	0.8 (0.5–1.3)
	Wage/casual labour	38 (13%)	40 (14%)	0.9 (0.5–1.5)
	Livestock/agriculture	41 (14%)	23 (8%)	1.8 (1.0–3.4)
	Other	92 (31%)	102 (35%)	0.8 (0.5–1.3)
Received any aid in last 3 months	Yes	28 (10%)	21 (7%)	Reference
	No	266 (90%)	269 (93%)	0.7 (0.4–1.3)
Largest HH expenditure on food	Yes	193 (65%)	193 (66%)	Reference
	No	104 (35%)	101 (34%)	1.1 (0.8–1.6)
Asset score	0 (lowest)	177 (57%)	157 (52%)	Reference
	1	39 (13%)	44 (15%)	0.8 (0.5–1.3)
	2	30 (10%)	24 (8%)	1.1 (0.6–2.0)
	3 (highest)	65 (21%)	75 (25%)	0.8 (0.5–1.2)
**Education**				
Attends school				
Yes		115 (62%)	159 (93%)	Reference
No		70 (38%)	12 (7%)	8.5 (4.3, 16.9)
Type of school				
Pre primary		93 (80%)	115 (72%)	Reference
Primary		23 (20%)	44 (28%)	0.5 (0.3,0.9)
School grade				
1		32 (82%)	52 (72%)	3.3 (1.0–10.0)
2+		7 (18%)	20 (28%)	Reference
Number of days missed				
<10 days		20 (43%)	16 (34%)	Reference
10 days plus		26 (57%)	31 (66%)	0.5 (0.2–1.3)
**Health**				
Child received any vaccinations				
Yes		293 (97%)	288 (98%)	Reference
No		9 (3%)	7 (2%)	1.3 (0.5–3.5)
Is child sick today				
Yes		24 (8%)	27 (9%)	Reference
No		283 (92%)	272 (91%)	1.1 (0.6–1.9)
Took action if child sick				
Yes		119 (83%)	112 (84%)	1.2 (0.6–2.2)
No		25 (17%)	21 (16%)	Reference

Children with disabilities were more likely to have their food prepared differently (OR = 2.1, 95% CI 1.1–4.2) and to experience difficulties in feeding (OR = 1.9, 95% CI 1.2–3.1) than neighbour controls (Table 3). The children with disabilities were also more likely not to be able to feed themselves (OR = 6.5, 95% CI 3.3–12.7). Almost all children (>98%) had been breastfed and there were no differences in breastfeeding patterns (ever/never, duration, current) between cases and controls, nor were there differences in the number of times that children had been fed per day or types of food eaten (data not shown).

**Table 3:** Relationship between disability and with feeding practices.

		Child with disabilities N (%)	Neighbour control N (%)	Age and sex adjusted OR (95% CI)
Food differently prepared	Yes	29 (10%)	14 (5%)	2.1 (1.1–4.2)
	No	272 (90%)	269 (95%)	Reference
Does child feed him/herself	Never/sometimes	55 (18%)	13 (4%)	6.5 (3.3,12.7)
	Always	249 (82%)	284 (96%)	Reference
Difficulty feeding	Yes	59 (19%)	32 (11%)	1.9 (1.2–3.1)
	No	252 (81%)	268 (89%)	Reference
Child breastfed	Yes	304 (98%)	297 (99.7%)	Reference
	No	6 (2%)	1 (0.3%)	6.4 (0.8–53.7)
Child still being breastfed	Yes	34 (11%)	28 (10%)	Reference
	No	274 (89%)	265 (90%)	0.8 (0.4–1.3)

Children with disabilities had lower MUAC for height and weight for height than neighbour controls, as well as lower weight for age, height for age and BMI for age (Table 4). These differences were also apparent between children with disabilities and their sibling controls, although the difference in weight for height did not reach statistical significance.

**Table 4:** Assessment of the relationship between anthropometric measures and disability.

		Cases	Controls			
Anthropometric measure*		Child with disabilities	Sibling controls	p-value**	Neighbourhood Controls	p-value**
MUAC for height***	N	155	113		165	
	Mean (sd)	-1.4 (1.3)	-1.0 (1.0)	0.003	-0.9 (1.0)	<0.001
Weight for age	N	294	183		287	
	Mean (sd)	-2.1 (1.6)	-1.5 (1.3)	<0.0001	-1.2 (1.4)	<0.0001
Height for age	N	225	179		282	
	Mean (sd)	-1.4 (1.8)	-1.0 (1.7)	0.02	-0.7 (1.9)	<0.0001
BMI for age	N	227	179		288	
	Mean (sd)	-1.6 (1.3)	-1.3 (1.1)	0.02	-1.3 (1.1)	0.002
Weight for height***	N	120	112		153	
	Mean (sd)	-1.5 (1.4)	-1.2 (1.1)	0.08	-1.1 (1.1)	0.03

* Exclusions because the measures were outside of the appropriate range: MUAC for height n = 1; Weight for age n = 4; Height for age n = 18; BMI for age n = 9; Weight for height n = 5.

**Adjusted for age and sex

*** WHO reference standards available for children aged 5 years and under only

Children with disabilities were significantly more likely to have general malnutrition (low weight for their age), stunting (low height for age) and have low BMI or low MUAC for age in comparison to neighbour controls or family controls, with odds ratios ranging from 1.6–2.9 (Table 5). Children with disabilities were almost twice as likely to have wasting (low weight for height) in comparison to neighbour controls (OR = 1.9, 95% CI 1.1–3.2), but this difference was not apparent compared with siblings (OR = 1.5, 95% CI 0.8–2.7). These associations were not attenuated by adjustment for the presence of feeding difficulties (data not shown).

**Table 5:** Relationship between malnutrition and disability status.

Measures of malnutrition*		Child with disabilities N (%)	Sibling control N (%)	Age- sex adjusted OR (95% CI)	Neighbour control N (%)	Age- sex adjusted OR (95% CI)
General malnutrition (low weight for age)	Yes	158 (54%)	63 (34%)	2.2 (1.5–3.2)	86 (30%)	2.7 (1.9–3.7)
	No	136 (46%)	120 (66%)	Reference	201 (70%)	Reference
Stunting (low height for age)	Yes	77 (34%)	42 (23%)	2.0 (1.4–3.1)	58 (21%)	1.8 (1.2–2.8)
	No	148 (66%)	137 (77%)	Reference	224 (79%)	Reference
Low BMI for age	Yes	84 (37%)	47 (26%)	1.8 (1.2–2.7)	68 (24%)	1.6 (1.0–2.4)
	No	143 (63%)	132 (74%)	Reference	220 (76%)	Reference
Wasting (low weight for height)	Yes	39 (33%)	26 (23%)	1.5 (0.8–2.7)	31 (20%)	1.9 (1.1–3.2)
	No	81 (67%)	86 (77%)	Reference	122 (80%)	Reference
Low MUAC for age	Yes	39 (25%)	17 (15%)	1.9 (1.0–3.6)	17 (10%)	2.9 (1.6–5.4)
	No	116 (75%)	96 (85%)	Reference	148 (90%)	Reference

* Defined as a z-score of -2 or less.

We assessed which characteristics were associated with a higher prevalence of general malnutrition (measured as low weight for age) among children with disabilities (Table 6). There were no clear correlates, except that children with disabilities not attending school had a higher prevalence of malnutrition than those attending school (OR = 1.7, 95% CI 1.0–2.8). There was also no variation in malnutrition by type of impairment (data not shown).

**Table 6:** Correlates of general malnutrition (low weight for age) among children with disabilities.

		Case with general malnutrition* N = 158	Case without general malnutrition N = 136	Prevalence of malnutrition (95% CI)	Age, sex adjusted OR
Age	<2	16	8	67% (47–82%)	Reference
	2-<5	48	44	52% (42–62%)	0.5 (0.2–1.4)
	5-<8	59	53	53% (44–62%)	0.5 (0.2–1.4)
	8+	35	31	53% (41–65%)	0.6 (0.2–1.5)
Sex	Boy	105	86	55% (48–62%)	Reference
	Girl	53	50	51% (42–61%)	0.8 (0.5–1.4)
Feeding difficulties	Yes	29	24	55% (41–67%)	1.1 (0.6–1.9)
	No	129	112	54% (47–60%)	Reference
Attend school	Yes	68	71	49% (41–57%)	Reference
	No	61	40	60% (51–69%)	1.7 (1.0–2.8)
Asset Score	0–1	103	100	51% (44–58%)	Reference
	2–3	55	36	60% (50–70%)	1.5 (0.9–2.5)

* Defined as a z-score of -2 or less.

### Sensitivity analyses

Data on armspan only was available for 51 children, while 712 had data on both arm span and height, and 29 had missing values for both measures. Children missing height measures were more likely to have physical impairments, compared to those with complete measures (p<0.001). The correlation between height and arm span was very high (0.97). When substituting arm span for height the differences between cases and both types of controls was strengthened for all the measures of malnutrition included in Tables 4 and 5.

## Discussion

This was a large population based case control study among children with disabilities, neighbour controls and sibling controls, conducted in a very poor area of Kenya which is experiencing protracted humanitarian food insecurity. Malnutrition was very common across all children examined in the study. There was clear evidence that children with disabilities were even more likely to be malnourished in comparison to both sibling or neighbour controls. This difference was evident whether malnutrition was measured in terms of stunting, wasting, low weight for age, low BMI or low MUAC. There was no difference in the types of food eaten between children with and without disabilities, but the quality and amount of food was not assessed.

Our findings on the link between malnutrition and disability need to be viewed within the context of other studies that have addressed this question. The UNICEF multiple indicator cluster survey (2005–06) including nearly 200,000 children across 15 countries showed that disability was significantly related to stunting in 5 of the countries, and to being underweight in 7 of the countries.[7] Differences were not apparent in the remaining countries. Other smaller studies show similarly mixed results. A case-control study in Nigeria showed that children with neurological impairments had more stunting (low height for age) and general malnutrition (low weight for age) compared to controls, but this difference was less apparent for children with other types of impairment.[8] A large case-control study conducted within an Indian slum found a link between malnutrition and disability, which was mediated by feeding difficulties.[6] In contrast, a study in Nepal did not demonstrate a link between disability with stunting or wasting overall, although children with motor milestone delay, and to a lesser extent those with learning difficulties, experienced more stunting.[9] Poor nutritional status is known to be linked to Cerebral Palsy and neurological impairment.[5,18–20] More research is therefore needed on this topic, given the sparse data and inconsistent results. It may be important to conduct future studies on more restricted age groups as different mechanisms may be more pertinent at different ages (e.g. stunting most relevant to reflect malnutrition at young ages).

The link between malnutrition and disability is multifaceted and there are several possible mechanisms.[3] Children with disabilities had more reported feeding difficulties. Children with disabilities were substantially less likely to be included in education and therefore were less likely to be included in school feeding programmes, which was the predominant route for targeting children of school age in the region. This is consistent with the finding that children of school-going age with disabilities had a lower prevalence of malnutrition than those not attending school. Neglect or lack of care for children with disabilities may also contribute towards malnutrition, though it was not assessed in this quantitative study. Malnutrition may also cause disability, as rickets was a relatively common form of disability and is caused by malnutrition. Children with disabilities were more vulnerable to malnutrition than their siblings, indicating that disability has a direct association with malnutrition, rather than through household poverty. However, given that the differences were greater between the child with disability and the neighbourhood control rather than a sibling control there may be household vulnerability to malnutrition.

Malnutrition was highly prevalent across all children examined in this study, even the controls, due to the chronic food shortages in the area. When the data were restricted to those aged less than 5 we see that general malnutrition was much more prevalent among cases (55%) sibling controls (35%) and neighbour controls (27%) than for children in Kenya as a whole (16%—UNICEF State of the World Report for Kenya).[21] Similarly, wasting was also more prevalent in our study samples (33%, 23%, 20%) than in Kenya overall (7%). These findings reflect the high level of food shortages in Turkana even compared with other regions in Kenya. Surprisingly, this difference was less marked for stunting (42%, 31%, 22% versus 35%), potentially because of the predisposition of Nilotic people in Turkana towards being tall which may partly offset the effect of food shortages on stunting.

In terms of other key findings, the study estimates that the minimum prevalence of moderate to severe disability in children of 0.75% (0.66–0.83%), and the dominant type was physical impairment. The true estimates are likely to be even higher, given that not all of the children with disabilities in the region would have been included. These figures are in line with the estimates from the key informant method study of childhood disability in Bangladesh and Pakistan, which estimated the prevalence of moderate to severe impairment of 0.9% in Bangladesh and 0.5% in Pakistan.[13] As in the present study, physical impairment was the most common type of impairment in both settings. In contrast, the World Report on Disability estimates that 5.1% of children have a moderate or severe disability, while 0.7% have a severe disability.[1] The discrepancy with our results is likely to be due, at least in part, to the threshold at which moderate/severe disability is defined as we likely used a more restrictive definition. The exclusion of children with disabilities from education is well established. [1, 22]

There were important strengths to the study. It was large and included population-based cases. The key informant method has been validated as a method for the identification of children with disabilities in the community. [14] Two sets of controls were included to assess whether disability was related to a higher prevalence of malnutrition at the community and household level, and therefore facilitated further speculation as to the nature of any association. The presence of disability was assessed through a questionnaire and confirmed through examination by a paediatrician. Detailed anthropometric measures were taken, as well as a comprehensive questionnaire assessment. Quality of the anthropometric and other data was ensured through one week of training, the development of hand-outs to support field activities, and close supervision in the field. Furthermore, there was assessment during the training to ensure that the fieldworkers were taking the anthropometric measures correctly, although this was not formally measured. In terms of limitations, the key informant method is unlikely to identify all children with disabilities in the community, and so will underestimate the prevalence. Data were collected on dietary intake, but not on quality of food, and these were collected through self-report rather than observation. There were missing data in a number of categories, but reasons for missingness were not recorded, and it was not possible to measure anthropometry (in particular height) for all children. Sibling controls could not be identified for all the cases (e.g. for cases that were only children) nor could neighbourhood controls always be found. Conditional logistic regression was therefore not undertaken, and we did not adjust for family or proximity as matching variables. This may have led to an underestimation of the difference in malnutrition between cases and controls due to clustering of malnutrition at the household and by proximity. It was not possible to include more sophisticated measures of anthropometry, such as DXA scans nor was their detailed assessment of the nature and severity impairment of the child. Standardised tools to assess intellectual impairment were not available that were relevant to the setting and so diagnosis was on the basis of the professional opinion of the paediatrician only. There are limitations to the use of a case-control design. Recall bias is possible and furthermore we cannot establish directionality or causality as the exposure and outcome are measured at the same time. The UNICEF-Washington Group disability questionnaire was developed through expert opinion and to date information is not available on its reliability and validity. Furthermore, although standardised questions were used where possible the validity and reliability had not always been assessed.

Our study findings point to several possible approaches to reduce malnutrition in children with disabilities in Turkana. First, there is widespread malnutrition for children in general in this setting and low receipt of aid, which needs to be addressed to improve the nutritional situation for all children, including those with disabilities. Improving nutritional status may also protect children from becoming disabled (e.g. due to rickets). There is also a need to focus on malnutrition among children with disabilities, given their vulnerability. One strategy is to promote the inclusion of children with disabilities in mainstream nutrition programmes (e.g. school feeding programmes) from which they may be excluded. Interventions focussing specifically on children with disabilities may also be needed. Such an approach is often used in high income countries where children with disabilities may receive specific interventions, such as tube feeding, to prevent or treat poor nutrition. These medical interventions may not be feasible in these poor settings, but other approaches such as routine screening of children with disabilities for malnutrition or training of caregivers in feeding practices may be appropriate. Finally, the malnutrition of the children with disabilities cannot be addressed in isolation from the other challenges that they face as they are closely inter-twined. In particular, the children with disabilities were more likely to be excluded from school and had large unmet needs for rehabilitation. These exclusions are contrary to the spirit of two key conventions relevant to children with disabilities: the UN Convention on the Rights of the Child, [23] and the UN Convention on the Rights of Persons with Disabilities,[24] and may increase vulnerability to malnutrition (e.g. through exclusion from feeding programmes). Promotion of inclusion, such as in health or schooling, among children with disabilities may therefore also reduce the prevalence of malnutrition. Future studies need to assess whether these findings are generalisable to other areas, and the evidence base on what works needs to be strengthened substantially in order to identify scalable interventions. [25,26]

## Conclusion

Children with disabilities are particularly vulnerable to malnutrition, even within an area of food insecurity where malnutrition was widespread across all children.
